# Significant beneficial effects of 12-weeks add-on yoga therapy on antipsychotic-stabilized schizophrenia patients through epigenetic modulation: novel findings from a randomized controlled study

**DOI:** 10.1192/j.eurpsy.2024.149

**Published:** 2024-08-27

**Authors:** M. Debnath, T. Mullapudi, R. Govindaraj, P. Raj, S. Varambally

**Affiliations:** ^1^Human Genetics; ^2^Neurophysiology; ^3^Psychiatry, National Institute of Mental Health and Neurosciences, Bangalore, India

## Abstract

**Introduction:**

Complementary and alternative therapy, especially yoga, is emerging as an important treatment modality for various complex disorders. Yoga therapy has reportedly been demonstrated to exhibit clinical benefits in schizophrenia. However, the modulatory effects of yoga therapy on the pathobiological pathways of schizophrenia are inadequately explored. Immune dysregulation is a widely recognized etiopathological construct of schizophrenia. It is not precisely known whether yoga therapy can modulate the expression of immune molecules by regulating gene expression and epigenetic processes in schizophrenia.

**Objectives:**

To understand the impact of 12-weeks add-on yoga therapy on the immune-inflammatory pathway in schizophrenia by examining plasma levels and gene expression levels of cytokines and complement proteins as well as by profiling promoter DNA methylation pattern of genes coding for cytokines and complement proteins.

**Methods:**

Fifty-seven schizophrenia patients fulfilling DSM-V criteria were recruited into the study and randomized into Yoga therapy (n=28) and waitlist control (n=29) groups. Plasma levels of IL-1β, IL-6, IL-10, IL-17, C1q, C2, C3, C4, C5, C5a, Factor B and Factor H by Multiplex Suspension Assay, quantification of gene expression of *Il1b, Il6, Il10, IL17, C3, C4* and *C5* genes by quantitative PCR and promoter DNA methylation of *Il1b, Il6, Il10, Il17, C3, C4* and *C5* genes by pyrosequencing were carried out in all the study participants.

**Results:**

Plasma levels of IL-1β (Z score= 2.42, p=0.02) dropped significantly and C2 (Z score= 2.24, p=0.03) levels increased after 12-weeks of yoga therapy. The expression of *Il1b* (Z score=2.45, p=0.01) and *Il6* (Z score=2.07, p=0.04) genes were significantly downregulated, while the levels of *C4* (Z score=2.23, p=0.03) gene was upregulated in schizophrenia patients of yoga therapy group. Two CpG sites in the promoter region of *Il1b* (all p≤0.05) and *Il6* (all p≤0.05) genes and three CpG sites in the promoter region of *C4* (all p<0.05) gene were hypermethylated, while two CpG sites in the gene body of *Il6* (all p≤0.05) gene and two CpG sites in the promoter region of *Il10* (all p ≤0.05) gene were hypomethylated after 12-weeks of yoga therapy in schizophrenia patients.

**Conclusions:**

Our findings provide important insights into the mode of action of yoga therapy in schizophrenia. This study for the first time reports the epigenetic effects of yoga therapy on immune-inflammatory pathway in schizophrenia.

**Disclosure of Interest:**

None Declared

